# In Vitro Evaluation of the Inhibitory Effect of Topical Ophthalmic Agents on *Acanthamoeba* Viability

**DOI:** 10.1167/tvst.8.5.17

**Published:** 2019-09-25

**Authors:** Wayne Heaselgrave, Anas Hamad, Steven Coles, Scott Hau

**Affiliations:** 1 Department of Biomedical Science & Physiology, University of Wolverhampton, Wolverhampton, UK; 2 Department of Biology, University of Anbar, Al-Ramadi, P.O. Box 55431, Ramadi, Iraq; 3 School of Science & the Environment, University of Worcester, Worcester, UK; 4 Department of External Disease, National Institute for Health Research-Moorfields Clinical Research Facility, Moorfields Eye Hospital, London, UK

**Keywords:** keratitis, treatment, *Acanthamoeba* spp.

## Abstract

**Purpose:**

To compare the antimicrobial effect of topical anesthetics, antivirals, antibiotics, and biocides on the viability of *Acanthamoeba* cysts and trophozoites in vitro.

**Methods:**

Amoebicidal and cysticidal assays were performed against both trophozoites and cysts of *Acanthamoeba castellanii* (ATCC 50370) and *Acanthamoeba polyphaga* (ATCC 30461). Test agents included topical ophthalmic preparations of common anesthetics, antivirals, antibiotics, and biocides. Organisms were exposed to serial two-fold dilutions of the test compounds in the wells of a microtiter plate to examine the effect on *Acanthamoeba* spp. In addition, the toxicity of each of the test compounds was determined against a mammalian cell line.

**Results:**

Proxymetacaine, oxybuprocaine, and especially tetracaine were all toxic to the trophozoites and cysts of *Acanthamoeba* spp., but lidocaine was well tolerated. The presence of the benzalkonium chloride (BAC) preservative in levofloxacin caused a high level of toxicity to trophozoites and cysts. With the diamidines, the presence of BAC in the propamidine drops was responsible for the activity against *Acanthamoeba* spp. Hexamidine drops without BAC showed good activity against trophozoites, and the biguanides polyhexamethylene biguanide, chlorhexidine, alexidine, and octenidine all showed excellent activity against trophozoites and cysts of both species.

**Conclusions:**

The antiamoebic effects of BAC, povidone iodine, and tetracaine are superior to the current diamidines and slightly inferior to the biguanides used in the treatment for *Acanthamoeba* keratitis.

**Translational Relevance:**

Ophthalmologists should be aware that certain topical anesthetics and ophthalmic preparations containing BAC prior to specimen sampling may affect the viability of *Acanthamoeba* spp. in vivo, resulting in false-negative results in diagnostic tests.

## Introduction

*Acanthamoeba* is a genus of small, free-living amoebae common to most soil and freshwater habitats.^1^ The organism has a life cycle of a feeding and replicating trophozoite, which, in response to adverse conditions, can form a dormant cyst stage.^1^ *Acanthamoeba* spp. are opportunistic pathogens of humans causing a fatal granulomatous encephalitis in the immunocompromised host and, more frequently, a potentially blinding keratitis in both noncontact lens or contact lens (CL) wearers.

Currently, there are approximately 4.1 million CL wearers in the United Kingdom,^2^ and established independent risk factors for developing acanthamoeba keratitis (AK) in CL wearers include exposure to tap water in the home,^3^,^4^ swimming or bathing when wearing CL,^4^,^5^ poor lens hygiene,^4^–^6^ and the use of rigid CLs in orthokeratology.^6^ Furthermore, previous outbreaks of AK in both the United Kingdom and United States and have been attributed to efficacy issue with certain CL disinfections system.^7^,^8^

Despite the sight-threatening risk with AK, in most series, it accounts for less than 5% of all CL-related microbial keratitis. The reported incidence rates in CL users are 1 to 2 per million in the United States and 17 to 20 per million in the United Kingdom.^4^ A recent study from a tertiary hospital in the United Kingdom reported an incidence rate of just 2.3% for *Acanthamoeba* over a 12-year period from over 1500 keratitis cases.^9^ Due to the small number of patients with AK, many are diagnosed late due to initially being misdiagnosed and treated for bacterial or other forms of keratitis, such as fungal and herpes simplex keratitis.^4^,^10^ A late diagnosis of AK has a massive impact on prognosis, and patients are more likely to develop poorer visual outcome, longer duration of treatment, corneal perforation, and the requirement of penetrating keratoplasty.^10^ Current medical therapy for AK is unlicensed and involves the topical administration of a biguanide, namely, 0.02% polyhexamethylene biguanide (PHMB) or 0.02% chlorhexidine, either as monotherapy or in combination with 0.1% propamidine or 0.1% hexamidine.^11^ PHMB and chlorhexidine have been reported to be the most effective and are effective against both trophozoites and cysts of *Acanthamoeba*.^12^–^14^

In the United Kingdom, the diagnosis of AK is not standardized and depends largely upon individual clinics and hospitals.^15^ A variety of methods can be used with culture of a corneal scrape on 2.5% nonnutrient agar that has been overlaid with a lawn of *Escherichia coli*, the most common method utilized and considered the gold standard. Despite the wide use of this culture-based method, poor sensitivity means that in many cases the culture comes back negative in patients with the infection. A recent study looking at diagnostic sensitivity reported a value of 33.3% for culture compared to 74.1% and 100% for polymerase chain reaction (PCR) and in vivo confocal microscopy (IVCM), respectively.^16^

One possibility for the low sensitivity of culture-based diagnostics for *Acanthamoeba* could be related to prior topical therapy, such as anesthetics and antibiotics, applied to the cornea prior to the corneal scrape being performed on the patient. Goldschmidt et al.^17^ have found that fluorescein and topical anesthetics could interfere with real-time PCR to detect herpesvirus and *Acanthamoeba*, resulting in false-negative results. Other studies have also shown that the use of Rose Bengal and Lissamine green reduced PCR detection rates for herpesvirus and toxoplasma.^18^ Furthermore, empirical antibiotic treatment with fluoroquinolone drugs or other biocides prior to diagnosis could have an effect on the viability of *Acanthamoeba*. Aside from the antimicrobial drugs, many ophthalmic preparations utilize benzalkonium chloride (BAC) as a preservative, and it has been shown that BAC is highly toxic to *Acanthamoeba*.^19^

Due to the potential effect of topical anesthetics and antimicrobials on the viability of *Acanthamoeba* cysts and trophozoites, we studied the activity of a range of commonly used topical anesthetics, antibiotics, antivirals, and biocides against the trophozoite and cyst stages of *Acanthamoeba polyphaga* (ATCC 30461) and *Acanthamoeba castellanii* (ATCC 50370) and a mammalian cell line.

## Materials and Methods

### Reagents and Test Compounds

We tested a range of ophthalmic preparations, including biocides, diamidines, anesthetics, antivirals, and antibiotics. All agents were obtained from Sigma Chemical Company Ltd. (Poole, UK) unless otherwise stated. The biocides, diamidines, and miscellaneous compounds included Brolene (propamidine isethionate 0.1% w/v; Sanofi, Guildford, UK), Desomedine (hexamidine di-isetionate 0.1%; Bausch & Lomb, Aubenas, France), PHMB (Lonza, Slough, UK), octenidine (Schulke & Mayr, Norderstedt, Germany), chlorhexidine digluconate 0.1% w/v, alexidine 0.1% w/v, propamidine 0.1% w/v, hexamidine 0.1% w/v, pentamidine 0.1% w/v, BAC 0.1% w/v, phenylmercuric nitrate 0.1% w/v, fluorescein sodium 2% w/v (Minims; Bausch & Lomb), and povidone iodine 5% w/v (Minims; Bausch & Lomb). Antibiotics used were preserved levofloxacin 5 mg/ml (Oftaquix; Santen, St Albans, UK), preservative-free levofloxacin 5 mg/ml (Oftaquix Unit Dose, Santen), moxifloxacin 0.5% w/v (Moxeza; Novartis, Frimley, UK), preserved chloramphenicol 0.5% w/v (Martindale Pharma, Brentwood, UK), and preservative-free chloramphenicol 0.5% w/v (Minims; Bausch & Lomb). The topical anesthetics tested were all in Minims formulation (Bausch & Lomb), and they included proxymetacaine (proparacaine) 0.5% w/v, tetracaine 1% w/v, oxybuprocaine 0.4% w/v, and lidocaine 4% w/v with fluorescein sodium 0.25% w/v. Antivirals used were trifluorothymidine (TFT) 1% w/v (Stockport Pharmaceuticals, Stockport, UK) and aciclovir 0.1% w/v. All the compounds were stored according to the manufacturers' recommendations.

### Test Organism Strains and Culture

*A. castellanii* (ATCC 50370) and *A. polyphaga* (ATCC 30461) were obtained from the American Type Culture Collection (LGC Standards, Teddington, UK). Trophozoites were maintained in a semidefined axenic broth medium as previously described.^20^ Cysts were produced using Neff's encystment medium (NEM) method as previously described.^20^ Trophozoites were seeded into large tissue culture flasks (Nunc, Altringham, UK) at a density of 1 × 10^5^ cells/ml in 50 ml of growth medium and incubated for 48 hours at 30°C. The trophozoites were harvested by centrifugation at 500 × *g* for 5 minutes and washed three times with one-fourth-strength Ringer's solution. The final pellet was then inoculated into 50 mL of NEM at a density of 1 × 10^6^ cells/ml into tissue culture flasks. The cultures were then incubated at 30°C for 7 days on a shaking incubator. The cysts were harvested for testing after 7 days of incubation in NEM and washed three times with one-fourth-strength Ringer's solution. The pellet was adjusted to 5 × 10^6^ cysts/ml by using a modified Fuchs Rosenthal hemocytometer (Hawksley, Lancing, UK), and the cysts were stored at 4 to 8°C for testing within 14 days.

### Amoebicidal Assays

In the trophozoite assay, serial two-fold dilutions of the test compounds were made in the wells of a tissue culture-grade microtiter plate (Helena Biosciences, Gateshead, UK). Ophthalmic preparation was used straight from the bottle and was serially diluted from the concentration stated on the product information label. Pure drugs were prepared as 1-mg/ml (0.1%) stock solutions in an appropriate solvent. Control wells received one-fourth-strength Ringer's solution in place of the test solution. Log-phase cultures of axenic trophozoites were adjusted to a concentration of 2 × 10^4^/ml in growth medium, and 100 μl of the calibrated suspension was added to the wells for incubation at 30°C in triplicate. After 48 hours, the wells were inspected using an inverted microscope. This was achieved by comparing the appearance of the trophozoites in the test wells to those in controls. Typically, this involves visually comparing the degree of amoeba growth relative to the control as well as looking for cell lysis and rounding of the amoebae. The minimum trophozoite inhibitory concentration (MTIC) was defined as 50% inhibition of *Acanthamoeba* trophozoite replication compared to the controls. The minimum trophozoite amoebicidal concentration (MTAC) was defined as the lowest concentration of test compound that resulted in the complete lysis or degeneration of the trophozoites.

### Cysticidal Assays

The cysticidal assay relies on the observation that *Acanthamoeba* cysts adhere to the well bottoms of the microtiter plates and remain attached following exposure to the test compound and removal by washing. The addition of live *Escherichia coli* to the wells, followed by incubation, results in encystment of viable cysts and replication of the emergent trophozoites. Serial two-fold dilutions of the test compounds were prepared with distilled water in the wells of the microtiter plate. Cysts were adjusted to a final concentration of 2 × 10^4^ cells/ml in one-fourth-strength Ringer's solution, and 100 μl was added to each well. The plates were then incubated at 30°C for 48 hours. After the incubation, the wells were aspirated to remove the drug using a Vacusip (Integra, Thatcham, UK) and refilled with one-fourth-strength Ringer's solution. This process was repeated three times to ensure removal of the drugs from the wells. After the final aspiration, the wells were filled with 100 μl of one-fourth-strength Ringer's containing live *E. coli* (ATCC 8739) at an optical density of 0.2 at 540 nm and incubated at 30°C. The minimum cysticidal concentration (MCC) was defined as the lowest concentration of test compound that resulted in no excystation and trophozoite replication after 7 days of incubation.

### Hep-2 Cell Cytotoxicity

The cytotoxicity of the test compounds was determined against the Hep-2 (HeLa derivative) human cervix carcinoma cell line (ECACC number 86030501) obtained from the European Collection of Cell Cultures (Centre for Applied Microbiology and Research, Salisbury, UK). The cells were grown and maintained at 37°C in minimum essential medium with 10% heat-inactivated fetal bovine serum (Life Technologies Ltd., Paisley, Scotland). Flasks containing confluent monolayers of cells were used to seed a 96-well microtiter plate at a concentration of 1 × 10^4^ cells/well in 100 μl of growth medium with incubation at 37°C. Once approximately 75% confluent growth occurred in the wells, the medium was changed, and the cells were used for cytotoxicity testing. Serial two-fold dilutions of the test agent in appropriate solvent were added to the wells, and the plate was incubated at 37°C for 24 hours. The degree of cytotoxicity was determined using the CellTiter 96 AQ_uous_ One Solution Cell Proliferation Assay (Promega, Southampton, UK). This is a colormetric assay in which metabolically active cells bioreduce a tetrazolium compound to generate a soluble colored formazan product whose abundance can be measured spectrophotometrically at 595 nm.^21^

### Transmission Electron Microscopy (TEM) of *Acanthamoeba* Cysts

For the TEM studies, Neff's cysts of *A. castellanii* were used. The cysts were exposed to the test formulations by using either topical ophthalmic preparations (tetracaine 1% and preserved chloramphenicol 0.5%) or a solutions made up to the same concentration used in ophthalmic preparations (PHMB 0.02%, unpreserved chloramphenicol 0.5%, BAC 0.05 mg/ml, and povidone-iodine 5%). Controls cysts were exposed to one-fourth-strength Ringer's solution. The cysts were exposed to the test formulations at 32°C for 1 hour. The agents were removed by washing the cysts with one-fourth-strength Ringer's solution and centrifuged at 1000 × *g* for 5 minutes. The resulting pellets were fixed with 2.5% (v/v) glutaraldehyde buffered with 0.1 M HEPES at pH 7.2 overnight at 4°C before being processed for TEM microscopy.

## Results

The activities of the test compounds against the trophozoites and cysts of *A. castellanii* (ATCC 50370) and *A. polyphaga* (ATCC 30461) and their toxicity for Hep-2 cells are shown in Tables 1 to 4.

**Table 1:** Efficacy of Topical Anesthetics and Fluorescein Sodium Against Acanthamoeba spp. Trophozoites and Cysts and Toxicity to a Human Epithelial Cell Line (Hep2)

Drug	In Vitro Drug Sensitivities (μg/ml) of:						
	*A. castellanii*			*A. polyphaga*			Hep2
	MTIC	MTAC	MCC	MTIC	MTAC	MCC	MCT
Proxymetacaine	39	156	156	39	78	156	39
Tetracaine	9.75	19.5	39	19.5	39	78	156
Oxybuprocaine	31.3	250	125	15.6	125	250	125
Lidocaine (+fluorescein)	312	1,250	10,000	625	312	1,250	5,000
Fluorescein sodium (2%)	>10,000	>10,000	>10,000	>10,000	>10,000	>10,000	1,250

MCT, minimum cytotoxic concentration.

**Table 2:** Efficacy of Topical Antibiotics, Antivirals, and Preservatives Against Acanthamoeba spp. Trophozoites and Cysts and Toxicity to a Human Epithelial Cell Line (Hep2)

Drug	In Vitro Drug Sensitivities (μg/ml) of:						
	*A. castellanii*			*A. polyphaga*			Hep2
	MTIC	MTAC	MCC	MTIC	MTAC	MCC	MCT
Levofloxacin (Oftaquix)^a^	78	156	625	156	312	625	39
Levofloxacin (pure drug)	312	1250	2500	625	1250	5000	78
Moxifloxacin (Moxeza)	625	2500	2500	1250	2500	2500	156
Chloramphenicol (pure drug)	312	625	2500	312	625	1250	625
Chloramphenicol (generic)^b^	78	312	625	39	156	312	312
Aciclovir (pure drug)	63	125	>500	125	250	>500	31.3
TFT^c^	312	625	5000	625	1250	2500	156
BAC	3.9	7.8	15.6	1	1.95	7.8	31.3
Phenylmercuric nitrate	1.95	3.9	31.3	1	1.95	15.6	3.9

a Compound is preserved with BAC (0.005% w/v).

b Compound is preserved with phenylmercuric nitrate (0.002% w/v).

c Compound is preserved with BAC (0.02% w/v).

**Table 3:** Efficacy of Diamidine Compounds Against Acanthamoeba spp. Trophozoites and Cysts and Toxicity to a Human Epithelial Cell Line (Hep2)

Drug	In Vitro Drug Sensitivities (μg/ml) of:						
	*A. castellanii*			*A. polyphaga*			Hep2
	MTIC	MTAC	MCC	MTIC	MTAC	MCC	MCT
Propamidine (pure drug)	62.3	250	>500	250	500	>500	250
Propamidine (Brolene)^a^	7.8	15.6	500	15.6	31.3	250	31.3
Hexamidine (pure drug)	7.8	62.3	250	7.8	31.3	250	62.3
Hexamidine (Desomedine)	7.8	62.3	250	7.8	31.3	250	62.3
Pentamidine (pure drug)	62.3	250	>500	125	250	>500	125

a Compound is preserved with BAC (0.005% w/v).

**Table 4:** Efficacy of Biguanides and Povidone Iodine Compounds Against Acanthamoeba spp. for Trophozoites and Cysts and Toxicity to a Human Epithelial Cell Line (Hep2)

Drug	In Vitro Drug Sensitivities (μg/ml) of:						
	*A. castellanii*			*A. polyphaga*			Hep2
	MTIC	MTAC	MCC	MTIC	MTAC	MCC	MCT
PHMB	1	3.9	15.6	1	7.8	7.8	31.3
Chlorhexidine	1	3.9	31.3	1.95	15.6	31.3	3.9
Octenidine	1	1.95	7.8	0.5	1	3.9	1.95
Alexidine	1	1.95	3.9	1.95	7.8	7.8	1
Povidone iodine	7.8	31.3	15.6	7.8	31.3	7.8	125

The results for the topical anesthetics and fluorescein sodium are shown in Table 1. The inhibitory range against *Acanthamoeba* trophozoites for the anesthetics proxymetacaine, tetracaine, and oxybuprocaine were 9.75 to 39 μg/ml, whereas lidocaine produced no inhibition of growth until the 312- to 625-μg/ml range for both species. In the trophozoite amoebicidal studies proxymetacaine, tetracaine, and oxybuprocaine were amoebicidal in the 19.5- to 250-μg/ml range, whereas with lidocaine, the amoebicidal activity against *A. polyphaga* and *A. castellanii* was 312 and 1250 μg/ml, respectively. For the cyst assays, proxymetacaine, tetracaine, and oxybuprocaine were cysticidal in the 39- to 250-μg/ml range. With lidocaine, the cysticidal activity against *A. polyphaga* and *A. castellanii* was 1.25 and 10 mg/ml, respectively. In the toxicity assay against the mammalian cell line, proxymetacaine, tetracaine, and oxybuprocaine were cytotoxic in the 39- to 156-μg/ml range, whereas lidocaine produced no cytotoxicity until 5 mg/ml. The lidocaine Minims contains fluorescein sodium and so as a control this was tested separately and found to be nontoxic at the 2% concentration (Table 1).

The results for the topical antibiotics, antivirals, and preservatives are shown in Table 2. For the fluoroquinolones, the levofloxacin (Oftaquix) formulation demonstrated trophozoite inhibitory and amoebicidal activity in the 78- to 312-μg/ml range, whereas for the unpreserved levofloxacin (pure drug without BAC), the values were in the 312- to 1250-μg/ml range for both species, a factor of 4 difference in magnitude from the commercial ophthalmic preparation. Moxifloxacin (Moxeza) demonstrated trophozoite inhibitory and amoebicidal activity in the 625- to 2500-μg/ml range for both species. Against cysts, levofloxacin (Oftaquix) was cysticidal at 625 μg/ml compared to levofloxacin (pure drug) and moxifloxacin, which showed cysticidal activity in the 2.5- to 5-mg/ml range. For the toxicity assay, levofloxacin (Oftaquix) and moxifloxacin gave a toxicity of 39 and 156 μg/ml, respectively. Two preparation of the chloramphenicol were tested. The preserved and preservative-free versions showed inhibitory activity against trophozoites at 39 to 312 μg/ml, whereas for the cysts this ranged from 312 μg/ml to 2.5 mg/ml for the two species of *Acanthamoeba*, respectively. Phenylmercuric nitrate, which is the preservative used in chloramphenicol, was active against trophozoites in the 1- to 3.9-μg/ml range and against cysts in the 15.6- to 31.3-μg/ml range. BAC, which is commonly added to ophthalmic preparations as a preservative, was active against trophozoites in the 1- to 7.8-μg/ml range and against cysts in the 7.8- to 15.6-μg/ml range.

The antiviral TFT was active against trophozoites in the 312- to 1250-μg/ml range and against cysts in the 2.5- to 5-mg/ml range. Aciclovir ophthalmic ointment (Zovirax) could not be used due to the soft paraffin base and so a solution was prepared from the pure drug. Aciclovir was active against trophozoites in the 63- to 250-μg/ml range but showed no activity against cysts in the range tested.

The effect of the diamidines against *Acanthamoeba* trophozoites and cysts and the toxicity to the mammalian cell line is shown in Table 3. With hexamidine, the pure drug and the Desomedine formulation performed identically in all tests against both species and the mammalian cell line. With propamidine, the pure drug showed a trophozoite inhibitory effect in the 63- to 250-μg/ml range compared to the 7.8- to 15.6-μg/ml range for the Brolene formulation. For the amoebicidal activities, propamidine (pure drug) was in the 250- to 500-μg/ml range compared to the 15.6- to 31.3-μg/ml range for the Brolene formulation. Both the pure drug and Brolene formulation showed limited to no activity against the cysts of both species. In the toxicity studies, the propamidine (pure drug) showed limited toxicity at 250 μg/ml compared to 31.3 μg/ml for the Brolene formulation. Pentamidine showed almost identical activity to that of propamidine. Comparatively, BAC was more effective than any of the diamidines tested, showing activity against the trophozoites and cysts and a greater cytotoxicity to mammalian cells (Table 2).

The effect of the biguanide compounds and povidone iodine against *Acanthamoeba* trophozoites and cysts and the toxicity to the mammalian cell line are shown in Table 4. In the trophozoite assay, all of the biguanides demonstrated inhibitory activity in the 0.5- to 1.95-μg/ml range and amoebicidal activity in the 1- to 15.6-μg/ml range. For cysticidal activity, the biguanides ranged from 3.9 to 31.3 μg/ml; both octenidine and alexidine were comparable to PHMB in terms of antimicrobial efficacy. For the toxicity assay, the biguanides ranged between 1 and 31.3 μg/ml, with PHMB demonstrating the highest value (least cytotoxic). Povidone iodine was active against trophozoites in the 7.8- to 31.3-μg/ml range and against cysts in the 7.8- to 15.6-μg/ml range.

**Figure 1:**
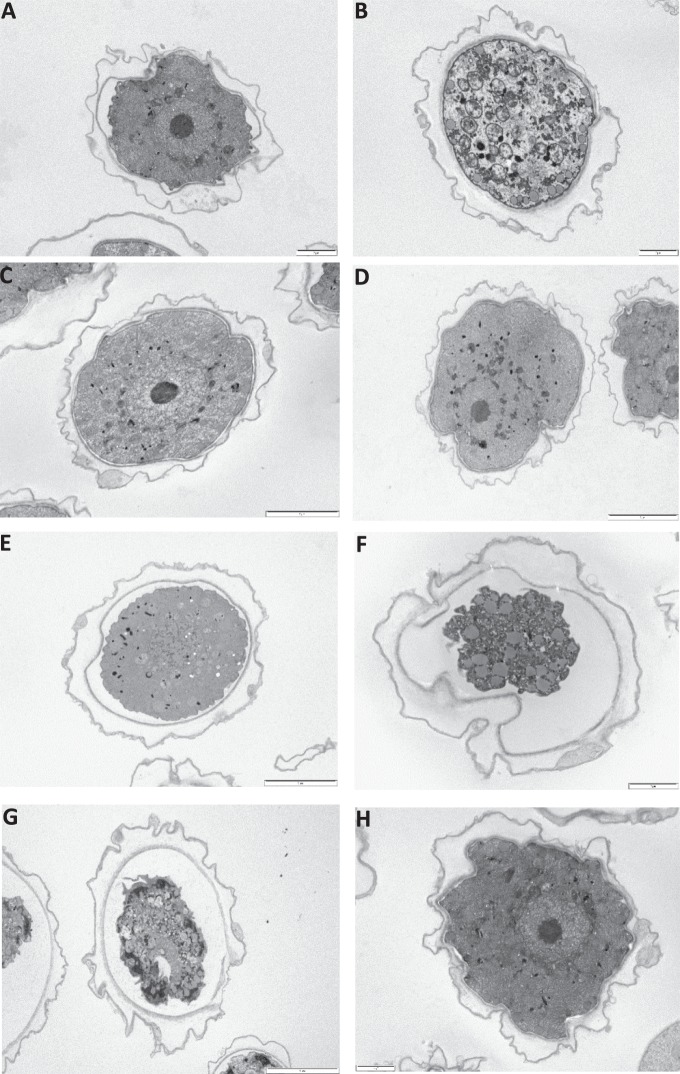
TEM images of Acanthamoeba cyst after 1-hour exposure to the following 7 agents: (A) untreated healthy cyst as control, (B) treated with 1% tetracaine, (C) treated with 0.5% preserved chloramphenicol, (D) treated with 0.1% propamidine pure drug, (E) treated with 0.05 mg/ml BAC, (F) treated with 5% povidone iodine, (G) treated with 0.02% PHMB, and (H) treated with 0.5% unpreserved chloramphenicol. Bar: 2 μm.

The changes in the morphology of the cysts when exposed to various compounds taken with TEM are shown in Figure 1. As a control, a healthy Neff's cyst in one-fourth-strength Ringer's solution can be seen in Figure 1A. The healthy cyst has a thick cyst wall surrounding the encysted trophozoite. The trophozoite plasma membrane touches the endocyst wall, taking up the full space available inside the cyst. In the cytoplasm, the nucleus can clearly be seen as can the rounded structures, including mitochondria and lysosomes.

After exposure to tetracaine, the nucleus is no longer visible and the cytoplasm is full of micelles caused by the breakup of the nuclear membrane (Fig. 1B). With preserved chloramphenicol, propamidine pure drug, and unpreserved chloramphenicol, no changes in the intracellular organization of the cytoplasm and nucleus were observed, respectively (Figs. 1C, 1D, 1H).

With BAC (0.05 mg/ml), the plasma membrane of the encysted trophozoite has been damaged and shrunk away from the walls of the endocyst and there is a lack of a defined nucleolus and an increase in the number of cytoplasmic micelles consistent with membrane damage (Fig. 1E).

With povidone iodine (5% w/v) and PHMB, the plasma membrane of the encysted trophozoite has been severely damaged and has shrunk significantly away from the walls of the endocyst. No defined nuclear structures were seen, and there are large numbers of micellar aggregations inside the cyst suggesting complete plasma membrane destruction in the encysted trophozoite (Figs. 1F, 1G).

## Discussion

AK is a sight-threatening corneal infection, and early diagnosis is paramount in achieving a better prognosis and visual outcome. Risk factor determination and clinical examination can often differentiate AK from other forms of keratitis, with ring infiltrate or disease confined to the epithelium to be more common compared to bacterial and fungal keratitis.^22^ However, in epithelial disease, especially associated with dendritic-type lesions, *Acanthamoeba* can be misdiagnosed with other causes of keratitis, such as herpes simplex (HSV) keratitis.^4^,^10^ Traditionally, cultures have been the mainstay in diagnosing *Acanthamoeba*, but a low culture-positive rate^23^ and prolonged incubation period often lead to a delay in diagnosis and treatment.^24^ The use of in vivo reflectance confocal microscopy and PCR in diagnosing AK have shown good promise, with sensitivity values ranging between 56% and 100%^25^–^28^ and 77% and 88%, respectively.^16^,^29^,^30^ The main advantages of IVCM are that it is noninvasive and it provides a rapid diagnosis, but the main limitations are the potential difficulty in differentiating pathogenic organisms from host cells and the diagnostic accuracy is dependent on observer experience.^26^ PCR testing is quicker and more sensitive than culture, returning a result often within days rather than weeks, but similar to cultures, false-negative results do occur. Possible factors include the amount of viable *Acanthamoeba* obtained from the corneal scrape or biopsy and an inhibition effect from the use of topical agents before microbiological sampling, such as prior empirical treatment with antibiotics, the use of anesthetics, and vital stains such as fluorescein.^17^,^31^ There are limited data on the inhibitory effect of topical anesthetics, with one study showing that proparacaine (proxymetacaine) did not adversely affect PCR,^31^ whereas in a second study,^17^ they found oxybuprocaine inhibited real-time PCR in detecting *Acanthamoeba*. We have found that the type of topical anesthetic greatly affected the viability of *Acanthamoeba* in that lidocaine had a much lower antimicrobial effect and, at therapeutic concentration, it did not exert significant antimicrobial activity against cysts and trophozoites for two species of *Acanthamoeba*. Although the lidocaine used in this study was combined with fluorescein, we did not find that testing fluorescein on its own had any major antimicrobial effect on the trophozoites or cysts. This contrasts with the other topical anesthetics, in particular tetracaine, which was observed to exert a much stronger antimicrobial effect against trophozoites and cysts of *Acanthamoeba* and toxicity to a human cell line. The antimicrobial effect of proxymetacaine was similar to oxybuprocaine for *A. castellanii*, but for *A. polyphaga*, proxymetacaine had a greater amoebicidal and cysticidal effect, in addition to being more toxic to the human cell line. These results suggest that the use of topical anesthetics, especially with tetracaine, can have a potent antiamoebic effect and it may be an important contributory factor in the reported low sensitivity for culture from corneal scrape.

Depending on the antibiotic protocol used, empirical treatment with third- or fourth-generation fluoroquinolones, due to their broad-spectrum antimicrobial activity, is often prescribed as an initial therapy for the treatment of microbial keratitis.^32^ We found that the pure drug of levofloxacin and a preservative-free preparation of moxifloxacin (Moxeza) did not exert any major antimicrobial effect on the viability of *Acanthamoeba*, whereas preserved levofloxacin (Oftaquix), which is preserved with BAC, had a much greater antimicrobial activity on both species of *Acanthamoeba*. This indicates it is the BAC in preserved levofloxacin rather than the drug itself that is causing the antimicrobial effect observed. Thompson and coworkers^31^ did not find any adverse effect on PCR amplification for *Acanthamoeba* with gatifloxacin or moxifloxacin. The gatifloxacin used in their study (Zymar; Allergan, Irvine, CA) was preserved with BAC, whereas the moxifloxacin was self-preserved. Although they did not test BAC on its own, the minimal inhibitory effect found with both antibiotics suggests that the effect of BAC on PCR in detecting *Acanthamoeba* DNA may be less compared to the amoebicidal and cysticidal assay methods used in this study.

Comparing the two preparations of chloramphenicol, the preserved drug had a much higher in vitro activity against *Acanthamoeba*, suggesting the antiamoebic effect is related to the preservative phenylmercuric nitrate. That said, the TEM images did not show much difference in the morphology with the preserved and preservative-free version of chloramphenicol.

This difference in antiamoebic activity was also seen when comparing preserved propamidine (Brolene), a common over-the-counter anti-infective ophthalmic preparation in the United Kingdom, with the pure drug propamidine. Previously, it has been shown that the concentrations of BAC typically found in ophthalmic medicines are highly toxic to *Acanthamoeba* trophozoites. In the study by Tu et al.,^19^ exposing trophozoites to BAC concentrations in the 10- to 30-μg/ml range produced up to a 4.5-log reduction in viability over 6.5 hours. However, this present study is the first to observe the effect of BAC-containing preparations against the highly resistant cystic stage of *Acanthamoeba*. We found the MTIC, MTAC, and MCC for BAC were significantly lower than the concentration of the BAC present in both Brolene (50 μg/ml) and Oftaquix (50 μg/ml) ophthalmic preparations. Indeed, this study has demonstrated that the presence of the BAC preservative in propamidine (Brolene) eye drops is likely to be solely responsible for the observed antiamoebic activity. Two previous studies have reported MTAC values for propamidine of 5 to 25 μg/ml and 0.49 to 0.97 μg/ml, but in both studies they only used the benzalkonium-preserved Brolene without comparing it to propamidine pure drug.^33^,^34^ This is the first study that has compared propamidine (pure drug) against BAC-preserved propamidine (Brolene), and we found the MTAC range for Brolene (15.6–31.3 μg/ml) to be similar to the study found by Hay et al.^34^ and higher than the values found in the study by Elder et al.^33^ However, when tested in the absence of BAC, the MTAC was much higher in the 250- to 500-μg/ml range (Table 2). This confirms that the antiamoebic activity of Brolene is down to the presence of BAC in the formulation. In fact, the amount of BAC in Brolene (50 μg/ml) is much higher than the MTAC found in this study, typically 12.8 times greater than the concentration observed to inhibit trophozoites. Morphologically, the TEM images show clear evidence that BAC causes damage to the cysts, and this is in agreement with the findings from Sunada et al.^35^ who found destruction of the cytoplasmic elements and separation of the inner and outer walls when cysts were exposed to BAC.^36^ However, in their study, the TEM cyst images with BAC and propamidine showed similar level of destruction of encysted trophozoite, and this is due to the fact that the authors did not use pure propamidine and instead they used GoldenEye eye drops that contain 0.1% w/v propamidine preserved with 0.05% BAC.

Many multidose ophthalmic preparations utilize BAC as a preservative to prevent contamination of the formulation; therefore, the empirical use of BAC preserved eye drops prior to a diagnosis of AK is likely to have a negative impact on the viability of *Acanthamoeba* present on the cornea, which may further contribute to the reported low positive culture rate for AK from corneal scrapes. Aciclovir demonstrated greater inhibitory and amoebicidal effect on trophozoites compared to TFT, but neither drug appeared to be effective against the cysts. Aciclovir is commonly used to treat HSV keratitis, and as AK is often misdiagnosed as HSV, the potential inhibitory effect on *Acanthamoeba* trophozoites would need to be considered.

When the experiments were repeated with hexamidine (Desomedine), another diamidine commonly used in the treatment of AK, we found that this gave identical results to hexamidine (pure drug). This result is not surprising as Desomedine, unlike Brolene, does not contain any preservatives. Moreover, this study has examined the activity of three different diamidines with increasing alkyl chain lengths between the aromatic benzene rings. The observation that the six-carbon hexyl chain length compound hexamidine has superior activity to the propyl and pentyl diamidines is consistent with a previous study showing that diamidine antiamoebic activity increases with lipophilicity due to increased interaction with the *Acanthamoeba* lipid bilayer.^37^

The aim of this study was to assess the potential inhibitory effect of prior administration of commonly used topical agents on the viability of *Acanthamoeba* before microbiological sampling, but to obtain a sense of how potent these agents are, we also compared the in vitro susceptibility to current and potential new treatment for AK. The results obtained in this study for chlorhexidine and PHMB are consistent with previously published studies, with PHMB demonstrating superior activity to chlorhexidine.^31^,^36^ The standard topical PHMB used in treating AK is 0.02% (200 μg/ml), which is nearly 20 times the mean MCC for PHMB found in this study; the finding agrees with the general favorable in vitro sensitivities and clinical outcome with PHMB compared to other antiamoebic drugs.^15^ However, Sunada et al.^35^ did not find that their *Acanthamoeba* isolates had high in vitro susceptibility to PHMB. Furthermore, the TEM appearance of the cysts in this study seem to be more affected, with damage to the cyst plasma membrane clearly seen, compared to Sunada et al.^35^ who found mainly a loss of electron-dense material in the cytoplasm.^36^ We found both alexidine and octenidine demonstrated very good in vitro sensitivities, and in fact, octenidine was superior to PHMB for MTAC and MCC for both species of *Acanthamoeba*, although the toxicity against the mammalian cell line was comparatively higher. This is the first study that has reported the activity of octenidine against *Acanthamoeba* cysts and trophozoites. The results of this study on alexidine agree with a previous study that reported an MTAC of 10 μg/ml, although they observed a much higher MCC (100 μg/ml).^38^ The exact reason for this difference is unclear, but the concentration of cysts used in the Alizadeh et al.^38^ study was 100-fold greater at 1 × 10^6^ compared to 1 × 10^4^/ml used in this and other published studies. Neither alexidine and octenidine are currently used to treat AK in a clinical setting, but the favorable antimicrobial activity against both *Acanthamoeba* cysts and trophozoites, especially with octenidine, warrants further investigation, as they may be useful in patients who do not respond to standard antiamoebic treatment. Alexidine, at a concentration of 1.6 μg/ml, is incorporated in one CL solution (AMO RevitaLens OcuTec), and it has been demonstrated that alexidine has excellent activity against cysts of *Acanthamoeba*.^39^ Aside from Alexidine, the majority of the multipurpose CL solutions (MPS) utilizes PHMB alone or in conjunction with polyquaternium-1. The concentration of PHMB-containing MPS are in the order of 1 μg/ml, which is equivalent to the MTIC found in this study. Accordingly, the aim of these MPS is to provide effective disinfectant properties through the prevention and inhibition of pathogenic organisms on the CL or the CL case, and therefore, it is possible that the release of the biocide from the CL can exert an inhibitory effect on the viability of *Acanthamoeba* on the cornea. The uptake and release of biocides occur in all types of CLs, but the interaction is complex and varies with numerous factors; therefore, the potential inhibitory effect on *Acanthamoeba* with the release of biocide from CLs on the cornea is currently unknown.

We found that povidone iodine, as in other studies, demonstrated good in vitro activity on *Acanthamoeba* isolates, with clear signs of damage from the TEM images. In fact, the MCC activity was identical to PHMB and superior to both propamidine and hexamidine. Although povidone iodine is not routinely used prior to performing a cornea scrape, the potential inhibitory effect on the viability of *Acanthamoeba* must be bore in mind.

The main limitation of this study is that findings from in vitro sensitivities do not always correlate with clinical outcome.^15^ There are many factors to explain this. The cysticidal assay exposed the cysts to the testing compound and then reexamined them after 7 days for excystation and trophozoite replication. In a clinical setting, corneal scrape for culture is performed straight after the instillation of topical anesthetics or vital stain; therefore, a shorter assay period would be more representative. Although we tested two commonly used fluoroquinolones and chloramphenicol, prior empirical antimicrobial treatment for microbial keratitis varies with different institutions, so the in vitro sensitivities might be different with different antibiotics. In addition, the interaction of the epithelium with a drug and the penetration of it into the stroma would be different with different types of drugs. These are shortcomings with in vitro sensitivity and efficacy studies of drugs on pathogens. Notwithstanding this, the potential adverse effect on the viability of *Acanthamoeba* with drugs used during clinical examination to reduce pain or the prior treatment with antibiotics before obtaining tissue specimens for culture or PCR need to be considered.

In conclusion, the present work indicates that the use of proxymetacaine, oxybuprocaine, and tetracaine to reduce pain; ophthalmic preparations containing preservatives such as BAC; and the use of povidone iodine prior to specimen sampling may affect the viability of *Acanthamoeba* in vivo, resulting in reduced culture yield and an inhibition effect on PCR amplification.
